# “It Opens the Conversation and Makes You Feel Less Alone. . .”: The role of disclosure and social support in managing polycystic ovary syndrome (PCOS)

**DOI:** 10.1177/13591053261419962

**Published:** 2026-02-23

**Authors:** Noelle Citron, Carly Biderman, Kenzie Tapp, Jessica C. Kichler, Suzanne McMurphy, Stacey L. Williams, Kendall Soucie

**Affiliations:** 1 University of Windsor, ON, Canada; 2 East Tennessee State University, TN, United States

**Keywords:** social support, disclosure, polycystic ovary syndrome, health psychology, women’s health

## Abstract

Polycystic Ovary Syndrome (PCOS) is a common endocrine syndrome, affecting 8%–13% of women worldwide. It has been described as an isolating and stigmatizing syndrome, complicating avenues for disclosure. In this study, we explored how women with PCOS disclose their diagnosis to others and what factors influence their decision to do so. Through a reflexive thematic analysis of 28 interviews, we found that women first *broached PCOS with others*, which led to *the to building blocks of social support*, which then *maintained meaningful conversations* over time. Supportive interactions were beneficial, while unsupportive ones led to *dead ends*. Our study is one of the first to capture the decision-making process underlying the disclosure of PCOS within women’s social networks. It extends prior research by highlighting how women decide whether, when, and how to share their diagnosis while weighing the risks of stigma against the benefits of social support.

## Introduction

Polycystic Ovary Syndrome (PCOS) is the most common endocrine syndrome diagnosed in women, affecting 8%–13% of individuals worldwide (Teede et al., 2018). It has widespread impacts on quality of life, with symptoms clustering into reproductive, metabolic, and psychological domains. Reproductive symptoms include ovarian cysts, menstrual irregularities, possible infertility, and elevated testosterone levels, the latter of which leads to changes in hair growth patterns on the body (Teede et al., 2018). Insulin-resistance and obesity/weight gain are the primary metabolic concerns, increasing the risk of type 2 diabetes. Psychological concerns include anxiety, depression, poor body image, and quality of life, as symptoms are distressing to most women (Damone et al., 2019; Kogure et al., 2019).

Although PCOS is well-researched biomedically (Azziz and Adashi, 2016), its psychosocial impacts are only recently gaining attention. Early work in this area centered around the view that PCOS disrupts quality of life and feminine identity (Kitzinger and Willmott, 2002), a perspective that has remained entrenched in the literature over the past two decades. For instance, Williams et al. (2015) found that women with PCOS adjusted their life plans (e.g. pregnancy) and reported receiving limited support from those they were close to. In a follow-up study, Williams et al. (2016) confirmed that many women experienced inadequate support from medical professionals; however, they identified partners, pets, and social media communities as important sources of support that positively influenced their quality of life. However, Thorpe et al. (2019) and Ee et al. (2020) found that fear of judgment, shame, and sensitivity to others’ perceptions led to hesitancy to reach out to others, perpetuating the silence and stigma associated with PCOS (Williams, 2023). These mixed findings highlight the need to better understand the processes that contribute to disclosure decisions, and whether support is available, accessible, and effective for those with PCOS.

Several models exist in the literature that explain how people disclose stigmatized identities, including chronic health conditions. These models include the Disclosure Process Model (Chaudoir and Fisher, 2010) and the Disclosure Decision Making Model (DD-MM; Choi et al., 2016; Greene, 2009). The Disclosure Process Model (Chaudoir and Fisher, 2010) views disclosure as continual and shaped by the individual’s goals, such as wanting to approach or avoid others, with avoidance often linked to perceived stigma, as well as by the disclosure event and the post-disclosure outcomes. In contrast, Greene’s (2009) *DD-MM* emphasizes how individuals assess risks when deciding whether to disclose stigmatizing personal information. It involves evaluating risks, making decisions based on internal and external factors, anticipating possible outcomes, and then executing (or withholding) the disclosure. Choi et al. (2016) extended the *DD-MM* to invisible conditions, emphasizing pre-disclosure planning, symptom unpredictability, stigma, and coping. Together, these models imply that the availability and quality of support for stigmatized individuals may depend not only on features of the stigmatized condition itself, but also on the strategies used to broach disclosure and support-seeking. This resonates with prior work showing that different ways of seeking support can shape others’ responses and that stigma-related avoidance is often paradoxically linked to greater rejection (Williams et al., 2016; Williams and Mickelson, 2008).

Social support often motivates disclosure and plays a crucial role in chronic illness management. Higher social support has been associated with better physical functioning, decreased illness related mortality, and better self-management of symptoms (Feldman et al., 2020). Crowley and Jackl (2023) found that direct, face-to-face disclosures were more likely to elicit supportive responses, with indirect strategies being met with less supportive responses (Williams and Mickelson, 2008). Direct disclosures have been associated with more engaged and active responses from listeners (Cipollina et al., 2022), as real-time sharing allows both parties to clarify and adapt the conversation, enhancing support (Steuber and High, 2015). Notably, the quality of social support from healthcare providers, family, and peers has been identified as a vital factor in treatment self-management in women with PCOS (Bazarganipour et al., 2017). Moreover, digital and peer-based support platforms also shape support experiences and coping processes (Gomula et al., 2024; Holbrey and Coulson, 2013).

Little is known about how disclosure and social support intersect in PCOS, however, given its unique challenges. Unlike many chronic conditions, PCOS is based in reproductive and hormonal health, areas often stigmatized and misunderstood in society (Williams, 2023). Symptoms such as infertility, menstrual irregularities, and appearance changes may make disclosure both sensitive and unpredictable, heightening fears of judgment and/or dismissal. Understanding these dynamics may help to explain why support experiences are often mixed while also highlighting opportunities to improve care and interpersonal understanding. As such, the following research questions guided our qualitative inquiry: (1) How do women with PCOS disclose their diagnosis to others in their lives, (2) What factors influence their decision to do so; and (3) How does social support shape their disclosure process?

## Methods

### Participants

Following institutional ethics clearance, 28 women diagnosed with PCOS in Canada participated in the present study (see Table 1). Participants ranged in age from 19 to 43 (*M* = 28.17, SD = 6.03), with an average time to diagnosis of 3.03 years. The sample consisted of Caucasian women (67%), followed by South Asian (7.14%), Latin American (7.14%), Black (3.6%), Arabic (3.6%), and Other (10.7%). Eligible participants were required to be Canadian citizens or permanent residents of Canada, English-speaking, over the age of 18 years, and diagnosed with PCOS by a medical provider. Participants were also required to have disclosed their PCOS diagnosis to at least one person within their social network (e.g. family members, romantic partners, and/or friends).

**Table 1.** Participant demographic information.

ID #	Age	Ethnicity	Relationship status	To whom they disclosed
1	23	Jewish	Single	Parents, siblings, and friends
2	26	White	Partnered/non-married committed relationship	Partner, mother, friends, online
3	28	White	Married/Civil Union	Partner, friends, mother, extended family
5	33	South Asian/Irish	Casually dating	Parents, friends
6	26	White	Single	Coworkers, friends
7	22	White	Married/Civil Union	Parents, sibling, partner, friends
8	21	Arabic	Single	Parents, siblings, friends
9	41	White	Married/Civil Union	Mother, friends, partner
10	31	Black/East Indian	Married/Civil Union	Partner, parents, siblings, extended family
11	28	White	Married/Civil Union	Friends, parents, siblings, husband
12	26	White	Casually Dating	Mother, siblings, friends
15	36	White	Partnered/non-married committed relationship	Mother, extended family, partner
16	30	Black	Single	Mother, sister, friend
17	30	White	Single	Parents, online
18	26	White	Married/Civil Union	Mother, partner
19	29	White	Single	Parents, coworkers, friends
20	22	White	Partnered/non-married committed relationship	Parents, siblings, friends
21	19	White	Single	Mother, friends
23	27	White	Married/Civil Union	Friends, partner
24	26	White	Partnered/non-married committed relationship	Parents, siblings, coworkers
25	37	South Asian	Married/Civil union	Parents, siblings, online
28	43	Latin Canadian	Married/Civil Union	Friends
29	32	White	Married/Civil Union	Parents, siblings, partner
30	29	White	Married/Civil Union	Parents, siblings, coworkers, extended family
31	31	White	Married/Civil Union	Parents, siblings, partner
32	27	Latin Canadian	Married/Civil Union	Parents, siblings, partner
33	21	White	Partnered/non-married committed relationship	Parents, friends
34	19	South Asian	Single	Parents, friends, online

*Note*. N = 28. Some IDs were removed (ID, 4,13,14,22, 26, 27) because participants did not attend the interview.

### Procedure

Participants were recruited across Canada via online advertisements, which were posted and circulated virtually through social media platforms (e.g. Facebook, Instagram) as well as in online PCOS support groups. A QR code was provided on the flyer, and potential participants were asked to scan the code to sign up to participate in the study. The code linked to a brief demographic screener to confirm eligibility. After participants completed the screener, the first author (interviewer) emailed them from a study-specific email address. She introduced herself and provided additional information about the interview. Those still interested were sent a virtual packet including an informed consent form, mental health and educational resources about PCOS, and a link to sign up for an interview time slot. All interviews were conducted virtually by the first author via Microsoft Teams.

All interviews were conducted in 2021–2022 during a single 60–90-minute session, with most interviews lasting approximately 1 hour. The semi-structured interview guide contained four domains related to PCOS: (1) PCOS diagnosis/lived experience, (2) disclosures, (3) influential disclosure experiences, and (4) social support (see Supplemental File). Participants then completed a 5-minute demographic survey in Qualtrics at the end of the interview to contextualize the sample. When the interview was complete, participants were compensated for their time with a $20 e-gift card to a place of their choice. Within 24 hours of each interview, Microsoft Teams provided a preliminary auto-generated verbatim transcript, which was stored in a password-protected folder only accessible to the research team. Transcripts were de-identified, assigned a unique ID code, and reviewed for accuracy by the first author.

### Data analysis

We analyzed the data using Braun and Clarke’s (2021, 2022) reflexive thematic analysis (RTA) approach. RTA is a method of identifying, organizing, and interpreting patterns of shared meaning across a data set. We chose this method because it connects the data to larger sociocultural belief systems, norms, and theoretical orientations, and values researcher subjectivity in all stages of the research process. We grounded this research within a social constructionist approach, which views knowledge as co-constructed through language, interaction, and context (Burr, 2003). We used an inductive, bottom-up approach aligned with an interpretative epistemology to remain grounded in participants’ lived experiences rather than applying a top-down framework that might constrain or overlook novel insights. This allowed us to both situate our findings in relation to existing models and highlight how the specific context of PCOS extends or challenges them.

RTA involves an iterative six-phase approach to data analysis: familiarization of the data, generating initial codes, constructing themes, reviewing themes, defining/naming themes, and writing up the results to reflect a coherent story. In Phase 1, we reviewed the transcribed interviews and familiarized ourselves with each transcript by (re)reading the interviews and noting personal reactions and observations. In Phase 2, we generated codes to outline areas of meaning within each transcript. The first and second authors coded the data, which involved individually constructing codes and then comparing them in biweekly meetings to ensure consistency. Disagreements were resolved through discussion, and the third author was an arbitrator during this process. Phases 3 and 4 involved combining conceptually similar codes from Phase 2 to form and label latent themes, as well as evaluating whether the theme correctly captured the meaning of the data. The first three authors then completed Phase 5, which was aimed at naming the themes and making sense of patterns in the data to form a coherent story. Finally, Phase 6 involved generating the report and locating exemplary quotes to illustrate the depth and breadth of the themes and subthemes.

We also engaged in multiple checks to ensure rigor (Tracy, 2010), including documenting each meeting via an audit trail and cross-referencing our notes (and transcripts when meetings were recorded) to foster an ongoing dialog at meetings. Moreover, we engaged in member reflections, which involved re-contacting participants and asking them to reflect on the themes that we constructed. Eleven participants responded and all reported that the themes reflected their experiences well (e.g. “this sums up what I have experienced in past conversations very well (both the good and the bad)”).

### Reflexive process

Our team is comprised of six White cisgender women (with the first author identifying as Middle Eastern Canadian), including three PhD candidates in Clinical Psychology, and three faculty members in the social sciences. Two authors have PCOS diagnoses, which shaped our insider perspectives, and enabled us to relate personally to participants’ narratives while also recognizing our position of interpretive power. Balancing empathy, curiosity, and personal reflection were a deliberate and ongoing part of our process. Our team regularly engaged in open dialog to reflect on how our social positions, professional experiences, diagnoses, and emotional responses influenced our analytic decisions, which ultimately enriched the depth and reflexivity of our analysis.

## Results

### Theme 1: Broaching PCOS with others

For many women, disclosing their PCOS diagnosis helped them explain or justify difficult symptoms. Disclosures were often framed around struggles with weight, fertility, hirsutism, and acne, with participants seeking understanding and validation.

#### Disclosing weight-related challenges

Weight fluctuations, a common symptom of PCOS, were often a key reason women disclosed their diagnosis. Talking about weight was difficult due to the stigma surrounding weight gain. Participant 1 described this challenge: “The weight stuff is like a little bit harder to talk about, just cause you know you can get defensive and like there’s nothing you can really do.” Others echoed similar concerns, stressing how hard it was for people to understand that their weight gain was not entirely within their control. Participant 16 further explained this problem:

It’s really hard to talk to people about [weight] to let them know, I’m not gaining weight because I’m eating a lot, I’m not gaining weight because I’m doing this, I’m gaining weight because I have a condition and it’s hard to treat it.

Supportive, non-judgmental conversations were particularly meaningful. Women appreciated when others listened, asked questions, and acknowledged their efforts without offering unsolicited advice. These interactions were especially empowering when participants retained agency over how much information to share. For example, Participant 19 had a productive conversation about weight with a co-worker who also had PCOS, stating that, “she’s kind of done the legwork. . . on the exercise regime she does, and she’s shared about the naturopath that she’s seen and what’s really worked for her. So, I found that was really helpful and a good source of support.”

In some cases, disclosure led to advocacy from close others. Participants valued when loved ones corrected misconceptions, especially among those less informed. Participant 29 shared an experience in which her mother corrected her grandparents’ views on weight:

My grandparents are an older generation, they’ll sometimes make comments about weight or something along those lines, and my mom constantly has to go back to them and go, “you need to remember she has something that’s causing [weight gain]”.

Such allyship offered reassurance and a sense of being understood.

Despite these positive experiences, not all disclosures were met with support. Some women encountered blame or shame when discussing their weight, leading them to withdraw from these conversations altogether. Participant 16 shared why she stopped turning to her mother, explaining that “it’s a negative conversation when I bring it up, so I choose not to go to [my mother] for support anymore.” Even well-meaning comments can be hurtful, leading some to establish clear boundaries. Participant 2 shared that she also no longer discusses weight with her mother: “We’re not going to discuss weight anymore because it comes across as degrading to me for her to say. . .you could stand to lose 15 or 20 pounds.” Despite the underlying intention, individuals should also “be aware that [their] words can be really hurtful to someone who’s been trying their best for so long” (Participant 2).

#### Sharing fertility struggles

For many women, disclosing their PCOS diagnosis was essential in explaining fertility struggles. These disclosures, often shared with romantic partners, family, or close friends served to manage expectations, seek support, and/or justify delays in having children. Reactions to these disclosures varied considerably; some responses fostered understanding and support, while others perpetuated societal pressures and misconceptions related to fertility. Women commonly shared their diagnosis with romantic partners to set realistic expectations about potential challenges in conceiving. For some, this disclosure was met with concern and fear about future fertility struggles. For instance, Participant 20 explained that “the reaction from [her] boyfriend was some fear about difficulties conceiving later on down the road.” Similarly, family members often inquired about plans for children, which led some participants to disclose their PCOS as a way of justifying their lack of children. For example, Participant 30 shared that she “told family members solely because they were like ‘so babies, when are we having babies’ and it’s just ‘no’.” By sharing their diagnosis, women hoped to curtail unwelcome inquiries and redirect conversations away from their fertility status.

Beyond managing expectations, many women found comfort in connecting with others who shared similar struggles. Participant 23 reflected on the importance of these connections:

When I started to have fertility issues, I met a lot of other mom friends and once we all kind of were talking about our experiences. . .we realized that so many of us had PCOS and it was a way for us to bond and connect and talk about it.

For some women, disclosing their PCOS became a natural part of conversations about fertility treatments. Participant 9 shared how discussions about her children often led to disclosing her diagnosis: “it is usually a part of disclosing that I did fertility [treatments] for my kids, and then they’ll say, ‘well why did you have to do fertility treatments?’. . .’oh I have PCOS,’ and they always nod their head. They had no idea what it means.” Such interactions highlight both the lack of public awareness about PCOS and the role of disclosure in educating others about the condition.

For some, disclosure was also a way to express the emotional burden of infertility. For Participant 23, being open about her diagnosis was a way to communicate her distress and seek understanding:

With the infertility especially, I wasn’t handling it very well. So, I think explaining to people and giving them full disclosure like, ‘I’m going through this, I’m having a hard time, this is what I have,’ and that’s kind of initially why I disclosed.

Participants reported that they appreciate responses that offer empathy, practical help, and/or space to simply talk. Most meaningful were moments where they felt seen beyond their diagnosis and were “still looked at as a person in that moment, not someone with a problem” (Participant 15).

#### Defending hirsutism and acne

Hirsutism and acne – two visible symptoms of PCOS – often served as entry points for disclosure. Described as “embarrassing” and “unfeminine,” these symptoms prompted women to justify their appearance. For instance, Participant 33 stated that she would “tell people almost as an excuse for having really hairy legs or like having really bad chest acne.” While acne was seen as more socially acceptable, hirsutism was harder to discuss:

I feel like acne is something that’s relatable to people my age like they can understand like oh yeah, I have acne too, whereas the hair growth on my face is more like I can only talk to PCOS friends about that (Participant 2).

For some, the presence of hirsutism and acne fostered productive conversations related to PCOS. Some women felt supported just by having others actively listen to their struggles with these symptoms. Participant 3 recalled continuously discussing her symptoms with her fiancé:

I tell him everything I’m like. . .“This chin hair, this situation,” and. . . now that he knows I can lean into, like, telling him like. . . this is like a PCOS thing. . . and then I’m, like, able to. . . talk openly about it. (Participant 3)

These positive and honest conversations about stigmatized symptoms helped to normalize them. Moreover, participants engaged in conversations that highlighted the intersectionality between cultural backgrounds and presentations of PCOS. Participant 8 recounted a time she reached out for support from a friend for her hirsutism:

Keeping in mind these, like artificially constructed standards about body hair, even the testosterone thing like uh, one of my other friends with PCOS, she is Black. . .and she was like, you know, like a lot of Black women naturally have higher levels of testosterone and, like, that doesn’t make them men. And I was like, oh well, that’s a good point.

When participants discussed these symptoms freely, it decreased some of the negative connotations surrounding them. These conversations gave women an opportunity to relate to others on both a gendered and cultural level.

### Theme 2: The building blocks of support

Positive first PCOS-related interactions with others often led to the construction of sustainable support systems and a reduction in perceived stigma with those specific individuals. Initial conversations about weight, fertility, hirsutism, and acne resulted in experiences that allowed women to feel supported and able to discuss the realities of their diagnosis. Additionally, these conversations led to a new and unexpected avenue of engagement and belonging through reciprocal disclosures, which were beneficial when the listener was non-judgmental, empathetic, and actively listening.

#### Reciprocal disclosures

For many women, sharing their PCOS diagnosis with others who also have PCOS promoted important dialog surrounding the syndrome. Often, when one woman disclosed her diagnosis, others felt comfortable sharing their own similar diagnoses. For instance, Participant 2 felt less alone when discussing her PCOS diagnosis with others sharing similar experiences: “That’s the first time I ever sat down with women that actually had PCOS too and had conversations with them and felt like I’m not the only person.”

Reciprocal disclosures were not limited to women with PCOS. Women with other reproductive syndromes shared their experiences as well. Participant 3 recalled how she felt when other women opened up to her: “I’ve told people and then they’re like, ‘I have it too,’ or ‘I have endometriosis’ and it’s, like, it just opens up, like, the conversation and makes you feel so much less alone.” Ultimately, these mutual experiences helped women to realize that there are others who understand their experiences, which fostered connection and mutual understanding.

#### Undiagnosed family members

Some participants discovered that family members had experienced PCOS symptoms but were never formally diagnosed. These symptoms often remained unspoken until participants disclosed their own diagnosis, prompting family members to reflect on and share similar experiences. This led to greater understanding within families, as participants felt more accepted when relatives could relate. One participant’s family tried to normalize PCOS for them by sharing:

“Oh, you know, your aunt has that.” And I was like, “Oh, I, I didn’t really realize.” I knew that she’d had fertility issues. And she has type 2 diabetes. And I guess looking back kind of has a lot of the, the visible signs and symptoms of PCOS. So, it probably should have been a given to me. (Participant 21)

#### Active, non-judgmental, and empathic listening

Participants reported that having someone actively listen without judgment and without providing unsolicited advice was the most impactful reaction to their disclosures. Giving advice may seem well intentioned; however, participants reported frustration related to unwanted or unsolicited advice. For instance, Participant 2 shared that “no matter who [she] tell[s], it’s like everyone wants to give [her] their opinion on how to fix it.” Conversely, Participant 32 reported that the disclosure experience that was most memorable and validating for her was one in which the audience actively listened in a non-judgmental way: “It’s like they’ve been impactful to me because they just listen. They don’t offer advice. They don’t, you know, make assumptions, they just listen and validate.”

A positive first conversation about PCOS may also impact a woman’s willingness to discuss their diagnosis with that person in the future. Participant 16 stressed the importance of initial disclosure experiences by outlining:

You can’t listen to react, you have to just listen and let the person talk and get things off their chest, and then maybe they’ll feel more comfortable coming to you again and talking to you about things that are a little bit more personal the next time.

Another important feature of active listening is expressing empathy. It is imperative for listeners to try to understand and be compassionate toward the daily experiences of women with PCOS. Many women did not expect others to offer solutions to their problems. Rather, they appreciated perspective-taking and empathy. Participant 2 described her expectations:

I know a lot of people think that when you come to them with an issue, they want you to fix it, but a lot of times they just want someone to listen, and they want someone who will kind of empathize with them.

### Theme 3: Maintaining meaningful conversations

Initial conversations around PCOS formed a basis for supportive relationships and deeper connections. These relationships were fostered in three ways: (1) when others demonstrated understanding by doing their own research on PCOS, (2) when others provided space to continue PCOS conversations over time, and (3) when participants felt a sense of community and connection with other women with PCOS.

#### Research by others

Participants reported that they felt supported when others showed interest in learning more about PCOS by doing their own research. Participant 7 recounted her partner showing support: “He. . . kind of took matters into his own hands and tried to do, like, an extent of his own research for how he could maybe help me.” Indeed, participants appreciated others’ initiative and interest in trying to understand PCOS on their own. Some listeners used their research as a means of being empathetic and understanding. For instance, Participant 23 expressed her deep appreciation toward her friends for researching the topic: “Everyone was really understanding and if they didn’t know what [PCOS] was, they researched it and – so that was really nice.”

The opposite also held true; it was damaging to relationships when listeners did not understand the condition and did not try to further their understanding of PCOS via research. Participant 5 recounted going to a weight loss clinic with her parents:

I brought up something about how they specialize in people with PCOS, and [my father] said, well, what does that have anything to do with it? So, I would say it would be fair to say he never went out and researched it. I don’t think my mom has either.

#### Evolving conversations over time

Another key component of maintaining meaningful conversations with others was recognizing that conversations around PCOS are often ongoing due to the chronic nature of the syndrome. Thus, having only one conversation is not sufficient to fully appreciate women’s experiences with PCOS. Participant 1 confirmed that “[she does not] feel like [she] very often disclose[s] everything at once, [she] feel[s] like it’s sort of in phases.”

Because more than one disclosure conversation is often needed to fully understand one’s experience, it is important for communication to remain open and consistent. After continuous positive conversations with a friend, Participant 16 expressed the following: “If I ever feel like I’m a little sad or I’m feeling extra bloated, or anything that kind of makes me feel negative about what’s going on with my body, I feel like I can talk to [my friend].” When women feel as though they can continuously discuss their PCOS with certain individuals, they tend to reach out to these specific connections for ongoing disclosures and support as needed.

#### Sense of community and connection with other women with PCOS

Many women felt safest disclosing their PCOS to other women, finding both comfort and understanding in these exchanges. Participant 2 confirmed that, “especially with women, [she] felt comfortable. . . [she] felt safe telling them and [she] also felt like it helped [her] explain a lot to them.” The comfort in telling women extended beyond close relationships, as participants also welcomed conversations with other women who had PCOS. Participant 8 shared that “there was a lot of relief associated with [disclosing], like it was this one sigh of relief and being like, ‘Oh I’m not alone.’” Indeed, women found consolation in the fact that other women had experienced similar struggles.

Some participants reported that engaging with online support communities was an accessible way to connect with others with PCOS. Participant 20 recounted that she “felt a little bit alone in [her] diagnosis so just being able to connect with other women virtually [she] thought was a really good platform.” Some women did not personally know other women with PCOS who they could have face-to-face conversations with; thus, virtual communities often proved to be a beneficial resource. Participant 25 expressed: “I just love the online support, and I think it’s important to just talk to people who have PCOS.” Connecting with other women through virtual platforms was a strong source of support for participants who wished to find others experiencing similar symptoms and stressors.

### Theme 4: Dead ends

Sometimes, conversations in which women disclosed their PCOS diagnosis did not prove to be fruitful or engaging. These disclosure experiences are labeled as “dead ends” because women often refused to seek support from the same person with whom they had a negative experience with.

#### Lack of awareness and understanding of PCOS

Many participants expressed ambivalence about having to repeatedly explain PCOS, especially given its high prevalence. While some felt people should already know about it, others acknowledged that “some people need an explanation because it’s still something that is not quite known” (Participant 30). Additionally, some participants compared it to other common chronic conditions that have more awareness around them: “When someone tells you they have diabetes, you’re like that must be difficult, that must change certain aspects of your life, but I don’t think when people hear you have PCOS that they think your life is any different” (Participant 3). Participants often expected at least basic awareness from others, especially other women, given the reproductive nature of the syndrome: “I think that speaks volumes. . . PCOS is so common, and I feel like it’s sad that women don’t really know it exists” (Participant 20).

Many significant male figures (e.g. fathers, brothers, romantic partners, etc.) struggled to understand the condition as well. Participant 7 justified her husband’s lack of knowledge of PCOS by sharing that, “he tries to and, you know, tries to be supportive the best he can, but [she] just [doesn’t] think he really understands purely because he’s a man.” For another participant, her husband’s lack of desire to understand the syndrome and support her drove them apart: “I think it was ultimately kind of what pushed us apart, because he didn’t want to learn, and didn’t want to help support me” (Participant 15).

When such disclosures are met with confusion, women may become more reluctant to try to explain their diagnosis to others. Participant 25 reflected on this idea: “I guess it comes back down to like trying to make my family and friends understand because they just don’t understand, and I think I’ve wasted too many hours or too many years trying for people to understand.” Reactions to disclosure mattered deeply, often shaping whether individuals felt safe or supported in sharing their diagnosis.

#### Lack of support

Dead end disclosure conversations occurred when listeners were not supportive of the PCOS diagnosis. Some participants expressed hopelessness related to their lack of support: “I don’t think I really have anyone; I don’t think there’s a lot of support for PCOS” (Participant 9), and “I don’t really have a support system in place for [PCOS]” (Participant 30). Often, a lack of support stemmed from negative reactions to participants’ initial disclosures. Participants reported that these types of interactions occurred with close others, such as family members and friends, and sometimes led them to stop communicating about their PCOS with specific individuals altogether. For example, Participant 16 described how lack of support started to affect her directly: “You wanna talk about [PCOS] and you wanna get some sort of support, and when you don’t get that support back, it just makes you not wanna talk about it at all.” Negative interactions not only shut down specific avenues for support with specific people, but they may also have more widespread impacts on disclosure avenues with others.

## Discussion

This study offers a novel contribution to the literature by examining the decision-making process underlying PCOS disclosures, including how women build, maintain, and sustain these disclosures within their social networks. We found that women navigated disclosure in nuanced ways, making careful decisions about when, how, and with whom to share their diagnosis, balancing the risk of stigma against the potential for support.

Most disclosure experiences began with participants carefully broaching PCOS to explain symptoms and connect with others. Early conversations often addressed weight fluctuations, infertility, hirsutism, and acne, which are symptoms consistently reported as distressing and socially challenging (Chrisler, 2011; Kitzinger and Willmott, 2002; Soucie et al., 2021). Women often felt the need to disclose their PCOS diagnosis to explain their visible symptoms (e.g. weight gain, hirsutism) and deflect personal blame. In contrast, women made deliberate choices whether to disclose their concealable symptoms (e.g. infertility, menstrual irregularities) that may otherwise remain silenced or hidden. This illustrates an important application in relation to the disclosure of visible versus concealable symptoms. These different disclosure demands explain why disclosure strategies varied across contexts, such as explaining acne to a coworker versus privately deciding whether to share fertility struggles with a partner.

Despite the stigma associated with PCOS, participants were motivated to discuss it with close others. These findings align with both autonomous and agentic disclosures that are common in other stigmatized conditions (Sharratt et al., 2020). Although participants experienced discomfort disclosing their diagnosis, they still initiated most conversations due to circumstance or necessity (i.e. autonomous disclosure). They were also in control of the narrative (i.e. agentic disclosure). These findings illustrate the deliberate and strategic nature of ongoing PCOS disclosures. Participants described, explained, and sometimes defended their symptoms to educate others and challenges misconceptions. They often clearly communicated their support needs and ended unproductive conversations.

Women in our sample most frequently disclosed their diagnosis to their family members, friends, and/or romantic partners. Disclosing to such individuals is common with other chronic conditions due to existing emotional bonds (Greene, 2009; Kaushansky et al., 2017). Such connections provide an explanation for why women seldom disclosed to people not as close to them, such as coworkers, unless there was a professional need to do so (e.g. symptom flareups). Two contradicting outcomes were possible when women provided explanations for their PCOS symptoms; they either experienced “shame and blame,” particularly regarding weight, or were believed and supported by others. Obesity on its own has been known to be accompanied by stigma (Puhl and Heuer, 2009) and aligns with recent literature illustrating that weight conversations are difficult to broach (Khan et al., 2018).

When others listened empathetically, participants felt able to share without judgment, which fostered understanding and connection, helping women feel supported (Barned et al., 2016; Steuber and Solomon, 2011). These positive interactions mitigated the risk of disclosing sensitive health information and led to increased trust and future reciprocity. This aligns with disclosure models that emphasize the role of listener reactions in shaping post-disclosure outcomes (Chaudoir and Fisher, 2010; Greene, 2009). It also reflects the paradox noted in prior work, where supportive responses are contingent on how stigmatized information is shared and what type of support is sought (Williams et al., 2016; Williams and Mickelson, 2008).

Maintaining supportive PCOS-related conversations over time involved three main components: (1) others researching PCOS to better understand it, (2) openness to ongoing discussion about PCOS, and (3) women being able to form a sense of community and connection with other PCOS diagnosed women. These are forms of *enacted* social support (Barrera, 1986), which are actions that aid in helping others, which can buffer stress and improve well-being (Feeney and Collins, 2015). Sustaining support over time is important, as ongoing validation and connection help women manage the chronic and often fluctuating challenges of PCOS. Women in our sample described that receiving instrumental support (i.e. researching the condition, sharing resources) helped them manage symptoms and feel supported. These accounts align with prior research indicating that social support can facilitate symptom management and contribute to improved quality of life (Earnshaw et al., 2012; Feldman et al., 2020).

However, when disclosure conversations did not go as expected, the audience became a *dead end*, and PCOS discussions were discontinued. Participants expressed frustration when close others made no effort to understand PCOS or offered unhelpful, judgmental responses. These unhelpful attempts at support are known as *miscarried helping*, which describes interactions in which others try to be helpful, but the help is perceived as negative and/or shame-inducing by the discloser (Tanaka et al., 2017).

As a whole, when the demands of a chronic condition like PCOS outweigh the available social support, women may experience a disturbance in their psychosocial adjustment (Presley et al., 2021). When their social support needs were not fulfilled by the people around them, women sometimes turned to peer-to-peer online communities. These spaces offered connection, validation, belongingness, and access to information that might not be readily available from in-person networks. However, the reliability of information varied, and some participants felt worse after visiting these sites, which aligns with past concerns about the medical validity and emotional risks of online support (Chiu et al., 2018; Soucie et al., 2021; Williams et al., 2015). Given that participants were recruited through social media, most were likely already engaged in online support. This is both a limitation (our findings may overrepresent women who are comfortable seeking help online), and a strength, as it sheds light on why digital spaces are appealing and how they benefit women with PCOS (Chopra et al., 2021; Gomula et al., 2024; Holbrey and Coulson, 2013). These platforms may lower barriers to disclosure, normalize experiences, and expand networks of validation, complementing offline support.

### Strengths and limitations

This study offers several contributions. To our knowledge, it is the first to examine PCOS disclosure experiences and their role in fostering social support. While prior research has applied a biopsychosocial perspective to PCOS, much of the literature continues to adopt a predominantly biomedical, deficit-based lens. Our study extends this work by demonstrating how disclosure processes and support responses intersect, situating PCOS within broader theories of stigmatized disclosure while showing where these models may fall short (e.g. reproductive health stigma, compounded symptoms, misconceptions). A unique strength of the present study is clarifying what women with PCOS find helpful in these moments of disclosure, which can guide others in how to respond effectively when disclosure occurs. This study extends these models and provides concrete guidance for improving support responses in practice. This contribution is important because it clarifies not only how women with PCOS seek and receive support, but also why support is often mixed or inconsistent.

We identify specific forms of social support that promote positive communication, offering a strength-based direction for future research. Future work could explore how disclosure processes differ for visible versus concealable symptoms of PCOS, and how fostering empathetic responses in clinical and social contexts might improve outcomes over time. Although our sample was relatively diverse concerning other intersections, a key limitation is the lack of socioeconomic and educational diversity in our sample. These factors may influence access to care, self-advocacy, and awareness of resources. Future studies should consider how intersecting social identities shape disclosure and support experiences, as this could illuminate disparities in who feels able to disclose, how support is mobilized, and where interventions are most needed. In closing, our study illustrates that thoughtful, informed support not only strengthens relationships, but also enhances psychosocial adjustment and self-management, which are critical outcomes for improving quality of life in women with PCOS.

## Supplemental Material

sj-docx-1-hpq-10.1177_13591053261419962 – Supplemental material for “It Opens the Conversation and Makes You Feel Less Alone. . .”: The role of disclosure and social support in managing polycystic ovary syndrome (PCOS) Supplemental material, sj-docx-1-hpq-10.1177_13591053261419962 for “It Opens the Conversation and Makes You Feel Less Alone. . .”: The role of disclosure and social support in managing polycystic ovary syndrome (PCOS) by Noelle Citron, Carly Biderman, Kenzie Tapp, Jessica C. Kichler, Suzanne McMurphy, Stacey L. Williams and Kendall Soucie in Journal of Health Psychology

## Data Availability

Given the sensitive and personal nature of the data collected during this qualitative study, the data set is not publicly available. Participants did not consent for the reanalysis of their de-identified transcripts by additional research teams, and they were assured that their data would remain confidential, de-identified and would not be shared beyond the research team. Sharing the data would the violate the terms of the ethical clearance granted by the University of Windsor’s review ethics board.
