# Supplementary material for: “It Opens the Conversation and Makes You Feel Less Alone. . .”: The role of disclosure and social support in managing polycystic ovary syndrome (PCOS)
Source: J Health Psychol. 2026 Feb 23;31(12):4828–42. doi: 10.1177/13591053261419962 (PMC13612881; doi:10.1177/13591053261419962)
Supplement: Supplementary file 1 [file sj-docx-1-hpq-10.1177_13591053261419962.docx]

**Semi-Structured Interview Guide**

Hi, thank you for your interest in our study, and for agreeing to participate. My name is Noelle and I’m currently a graduate student at the University of Windsor.

Would you like to tell me a little bit about yourself, so I am able to get to know you a bit better? Feel free to only share what you feel comfortable sharing.

Today I would like to talk with you about your experience living with PCOS and disclosing PCOS to at least one other person in your life. Since I am going to be asking you questions about your own diagnosis, I’d also like to share a little bit about mine. I also am a PCOS diagnosed woman and have been living with PCOS for about 5 years now. A lot of my research interests have come from my own experiences with the disorder, and it’s where the idea for the interview we’re doing today came from.

We’ll start by get your consent. As you know, the purpose of this study is to learn more about your experience with PCOS, specifically to learn about times you have told others about it and reached out for support. Your identity will not be revealed at any point and your responses will not be tied back to you. As we do the interview, please feel free to modify or decline to answer any questions; you also may suggest any questions you think I should be asking. If you need to take a break at any point, please let me know. This study has received ethics clearance from the University of Windsor REB.

Can you please confirm that you are willing to participate, and you consent to being video-taped (or audio recorded)? Do you consent to the use of anonymous quotations in the write up of the research? Please remember that you may withdraw from this study at any point during the interview and for any reason. Following the interview, you’ll have three weeks to withdraw your data for any reason.

Thank you! Do you have any questions for me before we begin? (also ask if they have any requirements, like needing extra breaks, etc.)

I am going to start the recording now.

**(General questions about PCOS).**

1. Can you tell me how you came to be diagnosed with PCOS? How old were you?
2. What has it been like for you to live with PCOS currently/(alt prompt: In what ways has PCOS affected your daily life?
3. Have you told anyone that you have PCOS? Who did you disclose your PCOS to?

**(Specific questions about disclosure):**

Now, I’d like to ask you a few questions about disclosure, so who you’ve told and why you decided to tell those people in your life**.**

4. Why did you decide to tell these people/this person (ask them to discuss each of the people they told and why they chose to tell them)?
5. How did this person/people react when you told them?
6. Did any of their reactions impact or influence you in any way?
7. Did anyone’s reactions surprise you? (Were their reactions as expected?)
8. How do you typically explain your PCOS to others?

**(Most memorable disclosure)**

Thank you for sharing these experiences with me. It helps me to understand who you told, why you told them, and how their reactions to your talking about your PCOS with them impacted you as well.

a. Of these experiences that you shared with me, what was your most memorable or impactful disclosure experience?
b. Why was this experience memorable?
c. Has this experience impacted how likely you are to reach out to this person again in the future?
d. Has this experience impacted the chances of you reaching out to others in the future?
e. *Additional prompts:* When did this happen/how old were you, what led up to it, what exactly happened (if unclear), what were you thinking/feeling during this experience, and how you react/respond? Finally, what was the impact of this experience on your life story/sense of self?

**(Social Support: Informal vs. Formal help-seeking avenues).**

I want to now turn to social support, and I want to ask you who you reach out to talk about PCOS.

10. Who do you typically reach out to for PCOS help and support? (alt: prompt: Have you reached out for support in any of the following ways:

- In person (friend, family, partner, close other)?
- Online/virtual communities?
- Community resource?
- Healthcare/mental health resource?
- Others?

11. Why did you choose to reach out for support from these resources or people?
12. Would you consider these resources or people trustworthy? Why?
13. Which/whom was the most helpful and why? Least helpful and why?
14. What would you like others (family members, friends, communities, etc.) to know about how to best support you?

**(Ending interview: long term concerns, and reflections).**

Thank you for describing who you reach out to for support and providing some great thoughts on what others should know to better support women with PCOS. The last section of this interview asks about any long-term concerns that you have about the future.

15. What impact do you expect PCOS to have on your future? What long-term concerns do you have?
16. If you could give someone who thinks they have PCOS advice, what would it be?

**(Final comments)**

Thank you for speaking with me today and for sharing your experiences telling others about your PCOS diagnosis, and what it’s been like for you to live with PCOS and manage it every day. I am grateful for your time today.

17. Is there anything that we have not covered in this interview that you feel is important for us to know?

Do you have any questions for me?

a. *Encourage participants to follow up via email if they have any additional questions, or if they forgot to mention something. (Keep an open dialogue)*
b. I will send an e-gift card for 25.00 of your choice, too.

**Member Checking:**

- Before we sign off, I’d like to ask you if you would be willing to be contacted again in about 4-6 months for the purpose of member checking. Member checking is a way for us researchers to know if the results we got from the data are accurate. We would send you a copy of some of the themes we are finding in the data, then you would fill out a brief survey to give us your opinion on our results.

We also ask that at this time I will leave the call and you can fill out a demographic data survey that I send the link to in the team’s chat. Your ID number is ______when it prompts you.

Thank you again!
